# Supplementary material for: Influence of preoperative frailty on quality of life after cardiac surgery: Protocol for a systematic review and meta-analysis
Source: PLoS One. 2022 Feb 4;17(2):e0262742. doi: 10.1371/journal.pone.0262742 (PMC8815968; doi:10.1371/journal.pone.0262742)
Supplement: S2 Appendix — (DOCX) [file pone.0262742.s002.docx]

| **#** | **Searches** |
| --- | --- |
| 1 | Cardiac Surgical Procedures/ or Thoracic Surgery/ |
| 2 | exp Cardiac Valve Annuloplasty/ or exp heart valve prosthesis implantation/ |
| 3 | (Valv* adj3 (repair* or reduc* or replace* or annuloplast* or insufficien* or regurgitat* or implant*)).ti,ab. |
| 4 | (TAVR or TAVI or TEER or edge-to-edge or mitraclip or mitral clip).ti,ab. |
| 5 | (Aortic adj3 (stenosis or insufficien* or regurgitat*)).ti,ab. |
| 6 | exp heart transplantation/ |
| 7 | ((Heart* or cardiac or valv*) adj3 (transplant* or Surger* or Surgical* or surgeon* or Operate? or Operating or Operation? or Presurger* or pre-surger* or Preoperat* or pre-operat*)).ti,ab. |
| 8 | exp Coronary Artery Bypass/ |
| 9 | (CABG or (coronary adj5 bypass)).ti,ab. |
| 10 | Heart-Assist Devices/ |
| 11 | (Artificial adj3 ventricle*).ti,ab. |
| 12 | ((Heart or ventricular or ventricle) adj3 assist adj3 device*).ti,ab. |
| 13 | ((Heart-assist or ventricular-assist or ventricle-assist or vascular-assist) adj3 device*).ti,ab. |
| 14 | or/1-13 |
| 15 | Geriatrics/ or exp Aged/ |
| 16 | (geriatric* or elder* or old age or advanced years or ageing or aging or gerontolog* or later life or youngest-old or middle-old or oldest-old or pensioner? or post-menopausal or postmenopausal or senior?).ti,ab. |
| 17 | ((old* or aged) adj1 (person? or people or adult? or man or woman or men or women or male? or female? or population)).ti,ab. |
| 18 | ((over or older) adj2 ("65" or "66" or "67" or "68" or "69" or "70" or "71" or "72" or "73" or "74" or "75" or "76" or "77" or "78" or "79" or "80" or "81" or "82" or "83" or "84" or "85" or "86" or "87" or "88" or "89" or "90" or "91" or "92" or "93" or "94" or "95" or "96" or "97" or "98" or "99" or "100") adj year?).ti,ab. |
| 19 | or/15-18 |
| 20 | Frail Elderly/ or Frailty/ or Sarcopenia/ |
| 21 | Geriatric Assessment/ or gait analysis/ or walking speed/ |
| 22 | (Frail* or sarcop?enia or functionally impair* or functional* impairment?).ti,ab. |
| 23 | (modified fried or fried phenotype or vulnerable elder? survey? or short physical performance battery).ti,ab. |
| 24 | (deficit accumulation adj3 index).ti,ab. |
| 25 | ((cardiovascular health study or CHS or MacArthur or successful ageing) adj3 scale).ti,ab. |
| 26 | or/20-25 |
| 27 | 14 and 19 and 26 |
| 28 | limit 27 to english language |
