# Supplementary material for: Influence of preoperative frailty on quality of life after cardiac surgery: Protocol for a systematic review and meta-analysis
Source: PLoS One. 2022 Feb 4;17(2):e0262742. doi: 10.1371/journal.pone.0262742 (PMC8815968; doi:10.1371/journal.pone.0262742)
Supplement: Attachment — Submitted filename: 20211028_Reponses.docx [file pone.0262742.s003.docx]

Dear Dr. Pasin,

Thank you very much for considering our manuscript for publication in the *PLOS ONE*. We have carefully read the Reviewers’ comments and addressed them to the best of our ability. We believe our manuscript was further improved by the review, and hope it will be deemed eligible for publication in the *PLOS ONE* at this time.

You will find our responses to each of the Reviewers’ comments below:

Reviewer #1:

**1. My suggestion is to try to find or establish an homogeneous definition of fragility in order to reduce bias.**

Thank you for this suggestion. We have edited the manuscript to try to clarify our accepted definitions of frailty. We have edited the manuscript to emphasize that currently there is no standard definition of frailty. There are multiple scores that have been developed to assess this syndrome, using a wide variety of difference clinical signs, symptoms, and tests to determine patient frailty.

Page 3; Introduction, lines 80-93.

*“The importance of frailty has been acknowledged by the American Heart Association (AHA) which has called for a better understanding of its role in cardiac care.^3^ There has been a call for trials to better improve the characterization of an older adult population and to evaluate the role of people living with frailty on clinical outcomes. However, frailty can be extremely difficult to quantify. While frailty does not yet have an internationally accepted definition, in general it is thought to be a heterogeneous syndrome that reflects multi-system dysfunction in which individuals are able to freely transition between severity states.^4-8^ Although it is more commonly encountered in an ageing population, it is a distinct entity from ageing. Multiple validated scores have been developed to attempt operationally define frailty in clinical practice. These include, but are not limited to, the Fried score, the Clinal Frailty Scale (CFS) and the Edmonton Frail Scale (EFS).^6, 9, 10^ However, there is no single accepted score that is universally used in clinical practice. In general, an assessment tool for frailty should be able to accurately identify frailty, reliably predict adverse clinical outcomes, predict response to potential therapies, be supported by biological causative theory, and be simple to apply.^4, 7, 11, 12^”*

Page 5; Methods, Frailty assessments, lines 129-133.

*“To be eligible, studies should report baseline frailty assessments, independent of the tool used.4, 6, 22, 23 Studies reporting frailty assessed only by biological (single or multiple) or radiological finding (i.e. sarcopenia on CT), single systems or clinical judgement alone will be excluded. Thus, only studies that utilize a frailty assessment that is multifaceted and considers multiple biological findings and/or organ systems will be included.”*

**Reviewer #2:**

**The authors performed a protocol for a systematic review and meta-analysis about frailty on quality of life after cardiac surgery.**

**The argument is potentially interesting but the manuscript is not to easy to read and it doesn't impact the clinical at the moment.**

We thank the Reviewer for the suggestions. We have taken great efforts to improve the clarity of the writing, and additionally, to emphasize the importance of the topic. We feel that the topic of frailty is extremely relevant for both clinicians who are deciding on eligibility for cardiac surgery procedures, as well as the aging patient demographic who need to decide on the best manner to treat their cardiovascular conditions. While other reviews have looked at the impact of frailty on mortality, there is a lack of understanding on how frailty impacts patient-centered outcomes, such as quality of life. For patients, often these outcomes are equally, if not more, important than mortality outcomes.

**1.** **page 5 line 106: why only 65 yo or older?**

We thank the reviewer for this question. Ageing is commonly measured by chronological age and, as a convention, a person aged 65 years or more is often referred to as ‘elderly’.(WHO 2010 definition of an elderly person). For this reason, we wanted to ensure our study was generalizable to older adults, and therefore chose this age, as it is the most accepted cutoff for defining an elderly population.

**2. line 107: did you exclude some interventions? otherwise is not necessary to specify.**

We excluded LVAD procedures and heart transplantation, as these procedures were fairly unique in terms of their procedural associated risk factors, and were also not particularly relevant for our population of interest. We have clarified this point in the manuscript in our Methods section.

*Page 11; Methods, Population, lines 124-130*

*“Cardiac surgeries that will be considered are coronary artery bypass grafting, valvular surgery and transcatheter valve interventions (transcatheter aortic valvular replacement (TAVR), thoracic aorta surgeries, and mitral valve repair (TEER). Left Ventricular Assist Device (LVAD) and heart transplantation procedures were not included, as it was felt* *these procedures were fairly unique in terms of their procedural associated risk factors, and were also not particularly relevant for our population of interest.”*

**3. line 111-113: is not clear the frailty assessment**

We thank the reviewer for this suggestion. We agree that the definition of frailty is difficult and nuanced. We have made several edits throughout the paper to emphasize the general consensus that the definition of frailty, as it stands currently, is poorly defined overall. However, several validated tools have been developed and studied to utilize in this regard. Thus, our study will make use of tools that are multifaceted and consider multiple biological findings and/or organ systems.

Page 3; Introduction, lines 80-93.

*“The importance of frailty has been acknowledged by the American Heart Association (AHA) which has called for a better understanding of its role in cardiac care.^3^ There has been a call for trials to better improve the characterization of an older adult population and to evaluate the role of people living with frailty on clinical outcomes. However, frailty can be extremely difficult to quantify. While frailty does not yet have an internationally accepted definition, in general it is thought to be a heterogeneous syndrome that reflects multi-system dysfunction in which individuals are able to freely transition between severity states.^4-8^ Although it is more commonly encountered in an ageing population, it is a distinct entity from ageing. Multiple validated scores have been developed to attempt operationally define frailty in clinical practice. These include, but are not limited to, the Fried score, the Clinal Frailty Scale (CFS) and the Edmonton Frail Scale (EFS).^6, 9, 10^ However, there is no single accepted score that is universally used in clinical practice. In general, an assessment tool for frailty should be able to accurately identify frailty, reliably predict adverse clinical outcomes, predict response to potential therapies, be supported by biological causative theory, and be simple to apply.^4, 7, 11, 12^”*

Page 5; Methods, Frailty assessments, lines 129-133.

*“To be eligible, studies should report baseline frailty assessments, independent of the tool used.4, 6, 22, 23 Studies reporting frailty assessed only by biological (single or multiple) or radiological finding (i.e. sarcopenia on CT), single systems or clinical judgement alone will be excluded. Thus, only studies that utilize a frailty assessment that is multifaceted and considers multiple biological findings and/or organ systems will be included.”*
